# The Effect of Mesenchymal Stem Cells on the Expression of IDO and Qa2 Molecules in Dendritic Cells

**DOI:** 10.15171/apb.2019.007

**Published:** 2019-02-02

**Authors:** Ali Moravej, Amin Kouhpayeh, Bita Geramizadeh, Negar Azarpira, Ramin Yaghobi, Yaser Mansoori, Mohammad-Hossein Karimi

**Affiliations:** ^1^Noncommunicable Diseases Research Centre, Fasa University of Medical Sciences, Fasa, Iran.; ^2^Transplant Research Center, Nemazee Hospital, Shiraz University of Medical Sciences, Shiraz, Iran.

**Keywords:** MSCs, DCs, IDO, Qa2, Immunomodulation

## Abstract

***Purpose:*** Mesenchymal stem cells (MSCs) have been shown to reduce the activity of immune cells, including dendritic cells (DCs). But the exact mechanism of mesenchymal inhibition of DCs is still unknown. In this study, the effect of mesenchymal cells on the expression of indoleamine dioxygenase (IDO) and Qa2 molecules in DCs was evaluated.

***Methods:*** MSCs and DCs were respectively isolated from the bone marrow and spleen of BALB/c mice. Then DCs were co-cultured with MSCs in the present and absence of lipopolysaccharides (LPS). Then the expression of mRNA and protein of IDO and Qa2 molecules were investigated in DCs that were treated with MSCs.

***Results:*** The expression of IDO and Qa2 mRNA in DCs that were treated with MSCs did not significantly differ from the control group. The expression of IDO protein in DCs that were cocultured with MSCs (in 1:10 and 1:50 ratios) in absence of LPS was increased, although they were not statistically significant (P values: 0.24 and 0.18, respectively). The expression of Qa2 protein in DCs that were co-cultured with MSCs (in 1:10 and 1:50 ratios) in presence of LPS was increased, although they were not statistically significant (P-values: 0.09 and 0.33, respectively).

***Conclusion:*** Our results denied the possibility that MSCs led to the induction of tolerogenic DCs by increasing the expression of the IDO and Qa2 immunomodulatory molecules.

## Introduction

Mesenchymal stem cells (MSCs) are multi-potent cells that are self-renewed under proper conditions.^1^ These cells are mainly in the bone marrow and their profession is to produce the cytokines needed to grow and multiply the hematopoietic stem cells.^2^ However, research suggests that MSCs can suppress the immune cells, thereby contributing in regulation of the immune system.^3-5^ For example, it is shown that these cells can reduce the activity of natural killer (NK) cells and neutrophils and their ability to produce cytokines.^3,4,6^ In addition, it has been revealed that MSCs can prevent maturation and differentiation of dendritic cells** (**DCs), resulting in a reduction in antigen presentation potency.^7-14^

DCs are one of the important immune cells that play important roles in stimulating and activating T cells. However, researches have shown that these cells can be transformed into tolerant cells under different conditions, and can inhibit T-cell lymphocytes with various mechanisms.^15-19^ For example, if an immature DC fail to become mature cell, and the rate of expression of MHC molecules and stimulant molecules (B7.1 and B7.2) does not increase at its surface, it will not be able to activate T lymphocytes, and could even convert T cells into T-reg cells.^19^ In addition, it has been shown that the expression of some compounds, such as indoleamine dioxygenase (IDO) and Qa2 molecules, in DCs can transform them into tolerogenic DCs that can inhibit the immune system, particular T lymphocytes.^20-22^

In various studies, it has been shown that HLA-G molecules in humans prevent the activation of the immune system.^23,24^ Observations show that HLA-G molecules can inhibit the Immune system in many ways. For example, these molecules inhibit the cytotoxicity of T-CD8^+^ cells and NK cells,^25^ prevent the proliferation of T-allo-reactive cells,^26^ inhibit antigen presenting cells (APCs) maturation,^27^ and lead to convert T cells to T-reg.^28^ The Qa2 molecule in mice is structurally and functionally similar to that of human HLA-G molecules.^21,22^ Studies in mice have shown that survival of skin transplanted tissue is more prolong in mice that produce higher levels of Qa2, due to decreased immune system strength.^20^ In general, the results of various studies indicate that the expression of the HLA-G molecule at the surface of human DCs and the expression of the Qa2 molecule at the surface of DCs in mice leads to tolerance of immunity.^20,21,28^

One of the mechanisms used by DCs to suppress T lymphocytes is using IDO. The IDO enzyme catabolizes tryptophan, which is an essential amino acid for the proliferation of T lymphocytes, thereby blocking the proliferation of T lymphocytes.^12,13,29^ Furthermore, it is shown that in a medium containing low level of tryptophan, due to the enzymatic activity of IDO, the phagocytosis capability of DCs is decreased. In addition to this study, there are numerous evidence that all indicate that increasing the expression of IDO in DCs can transform them into tolerogenic ones that can inhibit the immunity.^30-32^

Despite numerous studies on how MSCs inhibit the immune system, the exact mechanism of these cells’ function is not completely understood. So, further investigations, especially regarding the effect of MSCs on DCs, seems to be necessary. According to our knowledge, there is no published article on the effects of MSCs on the level of expression of the Qa2 and IDO immunomodulatory molecules in DCs. So, in this study, we tried to determine the effect of MSCs on the expression of Qa2 and IDO molecules on DCs.

## Materials and Methods

Monoclonal rat anti-mouse CD34 FITC-conjugated antibody, monoclonal rat anti-mouse Sca-1 PE-conjugated antibody, polyclonal rat anti-mouse CD11c PE-conjugated antibody, monoclonal rat anti mouse Qa2 antibody, monoclonal rat anti mouse IDO antibody, polyclonal rat anti mouse β-Actin HRP-conjugated antibody, monoclonal chicken anti rat HRP-conjugated antibody (all from Abcam, Cambridge, MA, USA), Dulbecco’s modification of Eagle medium (DMEM), Roswell Park Memorial Institute (RPMI), fetal bovine serum (FBS), enhanced luminol-based chemiluminescent substrate (ECL), Granulocyte-macrophage colony stimulating factor (GM-CSF), Trizol, RIPPA buffer, Bradford kit, protein inhibitor, polyvinylidene difluoride (PVDF) (all from Sigma, Ronkonkoma, NY, USA), cDNA synthesis kit (TAKARA, Japan), PCR SYBR Green kit (TAKARA, Japan), Nycodenz (Axis shield, Norway), Pan DC Microbead MACS (Miltenyibiotec, Germany).

### Isolation of bone marrow MSCs from Balb/C Mice

For isolation of MSCs, Balb/C mice, aged 4-5 weeks, were used. The mice were sacrificed by chloroform and the contents of their femur and tibia bones were removed by pressure using an insulin syringe was filled by DMEM. Isolated cells were placed in a cell culture flask, and were incubated in 5% CO_2_ and 95% humidity. After 24 hours, the supernatant was discarded and freshly medium was added to the cells attached to the flask. The cell culture medium was replaced every 24 hours, for about one month. The MSCs was passaged 5 times to be completely pure.

### Evaluation of the purity of MSCs based on surface markers

Mice MSCs lack CD34 but have Sca1 molecules on their surface. Therefore, to confirm the purity of MSCs, the presence of the specific markers on the surface of these cells was investigated. To this, 5 × 10^5^ of the MSCs were transferred to a flow cytometric tube and rat anti-mouse CD34-FITC conjugated antibody (1 μg/mL) and rat anti-mouse Sca1-PE conjugated antibody (1 μg/mL) antibodies were added. The appropriate isotype with each antibody was added to a separate tube. After 30 minutes of incubation in the dark at room temperature, the cells were washed 2-3 times with phosphate buffer saline (PBS) and then evaluated by flow cytometry. Total of 2 × 10^5^ events were measured during flow cytometry. Then, data was collected using the Cell Quest software and data analysis was performed with FlowJo version 7.6 software.

### Differentiation of MSCs into bone and fat cells

In order to assure the mesenchymal nature of the cells and to evaluate their differentiation ability, 1 × 10^5^ cells in passage 4 were cultured in 6 cm^2^ dishes containing DMEM with 15% FBS and 1% antibiotics. After 80% of the surface of the plate was occupied by the cells, the cells were replaced with differentiating media (osteocyte medium containing: 50 μg ascorbic acid 3 phosphate, DMEM, 10 nM dexamethasone and 10 mM beta-glycerol phosphate. Adipocyte medium containing: 100 nM dexamethasone, DMEM and 50 μg/mL indomethacin). Differentiation process was continued for 14 days. At the end of the second week, alizarin red staining for osteocytes, and oil red staining for adipocytes were done. For alizarin red staining, the cell layer was washed with PBS and fixed for 10 minutes with methanol and then stained with a solution of 1% Alizarin in ammonia 25% for 2 minutes. Then cells were washed with distilled water and after drying, microscopic evaluation was done. For oil red staining, the cells were fixed with 4% formalin at room temperature. Then cells were washed with 70% ethanol and stained with 5% oil red in 99% isopropanol for 5-15 minutes. At the end, cells were washed with 70% ethanol 3 times and observed with a microscope.

### Isolation of DCs

Balb/C mice were used to isolate DCs. After sacrificing the mice with chloroform, the spleens were removed from the left side of their body. The collagenase solution was injected into the spleens. The spleens were shredded with scissors and was placed in the incubator for 30 minutes until collagenase penetrate the spleen tissue. The spleens were then passed through the mesh. For each 5 × 10^8^ cells, 3 mL Nycodenz was poured into a Falcon tube, then a solution containing cells and RPMI medium was slowly added to Nycodenz. The tubes were centrifuged to form 2 phases. After centrifugation, a white ring, that contained white blood cells, was observed between the 2 phases. Cells were removed and added to a new Falcon tube. For each 1 × 10^8^ cells, 400 μL of MACS buffer (PBS-EDTA 2 mM) and 100 μL of Pan DC Microbead was added and placed at 8 to 4°C for 15 minutes. Then, for each 1× 10^8^ cells, 2 mL buffer were added and placed on the LS column of the MACS. Non-attached cells were discarded. The column was removed from the separator and placed on a new falcon. Five milliliters of buffer was added to the column and the piston gently pulled out the cells from the column and collected in a falcon. Tubes containing cell were centrifuged. The soup was discarded and the cells were dissolved in RPMI solution containing 10% FBS. To evaluate the purity of DCs, the presence of CD11c specific marker on the surface of these cells was evaluated using rat anti-mouse CD11c-PE conjugated antibody and flow cytometer. Total of 2 × 10^5^ events were measured during flow cytometry.

### Co-culture of MSCs with DCs

As shown in Table 1, DCs (2 × 10^6^ cells) and MSCs with ratios of 1: 10 and 1:50 in the presence or absence of lipopolysaccharides (LPS) in cell culture 6 wheel plates were co-cultured. After treating DCs with MSCs, they can easily be removed from the wells because MSCs are adherent cells so they stick to the bottom of the well, but DCs are in-adherent. Transwell cell culture plates were also used to examine the effect of MSCs secretion on DCs. DCs and MSCs with ratios of 1:10 and 1:50 in presence or absence of LPS were cultured in Transwell cell culture plates. It should be noted that in Transwell plates, there is a distance between the 2 cell groups that are cultured together, and the cells are not able to communicate directly with each other, and therefore only their secretions affect each other. In Transwell plates, DCs were cultured in lower chamber and MSCs were cultured in upper chamber. Also, GM-CSF at 50 ng/mL concentration was added to all culture plates as growth factor.

### Evaluation of IDO and Qa2 gene expression in DCs treated with MSCs by Realtime PCR method

After treating DCs with MSCs, about 2 × 10^6^ cells were transferred to the microtube, and 1 mL of Trizol and 250 μL of chloroform were added. The tubes were placed on ice for 5 minutes and then centrifuged for 15 minutes at 13 000 g. The upper phase was separated and added to the same volume of isopropanol. Then it was centrifuged for 15 minutes at 13 000 g at 4°C. Then 75% ethanol was added and after 10 minutes, the microtubes were centrifuged at 13 000 g for 10 minutes. At the end, 30 μL of distilled water was added to the precipitate. Then, the cDNA was constructed using a cDNA synthesis kit based on the kit instruction. Specific primers for the Qa2 gene (forward: 5ʹ- GCGGGTTGTAAAGTCCAC -3ʹ, reverse 5ʹ- AGAGCAGCATTGTTAGAGC -3ʹ) and IDO gene (forward: 5ʹ- GACAGCAATGGCACTCAG -3ʹ, reverse 5ʹ- CGTTCTCAATCAGCACAGG -3ʹ) and GAPDH (forward: 5ʹ- CGGTGTGAACGGATTTGGC -3ʹ, reverse 5ʹ-GTGAGTGGAGTCATACTGGAAC -3ʹ) (as internal control) was designed with the AlleleID software. To carry out the real-time polymerase chain reaction (PCR), the PCR SYBR Green kit were used and were performed according to the kit. In order to construct the cDNA by reverse transcription method, first, a cDNA mixture containing PrimeScript buffer, RNase inhibitor, PrimeScript RTase and RNase free dH2O were prepared and distributed in 200 μL microtubes. Then, 2 μL samples (0.5 μg RNA) were added to the microtubes. The microtubes were mixed and mini-centrifugated and transferred to a Thermal cycler apparatus. Temperature program included 30°C for 10 minutes, 42°C for 40 minutes, 70°C for 10 minutes was performed. To perform PCR by Syber green, PCR mixture containing SYBR Premix Ex Taq II, forward primer, reverse primer, ROX reference dye and dH2O was prepared, and distributed in 100 μL microtubes. Then, 2 μL of samples, equivalent to 0.1 μg of cDNA, were added to the microtubes. The microtubes were mixed and mini-centrifugated and transferred to a thermal cycler. Temperature program was included 95°C for 45 seconds, 58°C for 45 seconds and 72°C for 60 seconds. The change in genes expression relative to the GAPDH control gene in each sample was calculated using the 2^-ΔΔCT^ formula.

### Evaluation of IDO and Qa2 gene expression in DCs treated with MSCs by Western Blot

After treating DCs with MSCs, about 5 × 10^6^ of these cells were transferred to the microtubes and using RIPPA buffer, proteins were extracted and used to evaluate the expression of the IDO and Qa2 protein using the Western Blot method. For this purpose, for every 10 × 10^6^ cells, 300 μL of RIPPA buffer and 1% volume of protein inhibitor was added and incubated for 2 hours on ice in darkness. The samples were centrifuged for 15 minutes at 12 000 rpm to precipitate non-protein substances. Protein concentration was measured by Bradford kit and adjusted to 2 μg/μL. Then vertical electrophoresis of proteins was performed using sodium dodecyl sulfate polyacrylamide gel electrophoresis (SDS-PAGE) technique. For this purpose, the 12.5% resolving gel and 3% stacking gel was used. The amount of 30 μg of protein extracted from the cells were mixed with sample buffer and heated at 95°C for 2 minutes and immediately transferred on ice. Then, samples were loaded into the wells. Electrophoresis began at 60 V and after 30 minutes the voltage was increased to 100 V. Electrophoresis was performed for approximately 2 hours. Subsequently, transfer of proteins from gel to PVDF membrane was carried out using semi-dry western blot set (GE healthcare, Boston, MA, USA) with current of 1.5 mA/cm^2^ of gel for 90 minutes. Then detection of proteins was performed with monoclonal rat anti-mouse Qa2, monoclonal rat anti-mouse IDO and monoclonal rat anti-mouse β-Actin antibodies (as internal control. For this purpose, PVDF paper was placed in 96% methanol for 15 seconds and washed with buffer for 5 minutes. It was then placed in a blocking buffer for 1 hour at room temperature or for 12 hours at 4°C. It was then placed at room temperature in a blocking buffer containing diluted antibodies for 1 hour. Then, 5 washing rinses was performed for 7 minutes with a washing buffer. Then PVDF paper was placed in a blotting buffer containing monoclonal HRP-conjugated chicken anti rat secondary antibody for 1 hour. Then, 5 washings were performed for 7 minutes with a washing buffer. Subsequently, the visualization of the bands was performed by ECL substrate. For this purpose, 100 μL of the ECL substrate solution was placed on the surface of the PVDF membrane per 1 cm^2^ of the PVDF membrane for 5 minutes. Finally, protein bands were visualized in a ChemiDoc device (Bio Rad, Pleasanton, CA, USA).

**Table 1:** Study groups

**Groups**	**MSCs/DCs ratio**	**Co-culture**	**LPS**	**GM-CSF**
1	DCs only	-	-	+
2	1:10	Transwell	-	+
3	1:50	Transwell	-	+
4	1:10	Direct co-culture	-	+
5	1:50	Direct co-culture	-	+
6	DCs only	-	+	+
7	1:10	Transwell	+	+
8	1:50	Transwell	+	+
9	1:10	Direct co-culture	+	+
10	1:50	Direct co-culture	+	+

### Statistical analyzes

The data were analyzed using Tukey one way ANOVA test. *P* < 0.05 was considered statistically significant. Statistical analysis was performed using SPSS software.

## Results and Discussion

### Purification and proliferation of MSCs

**Figure 1:**
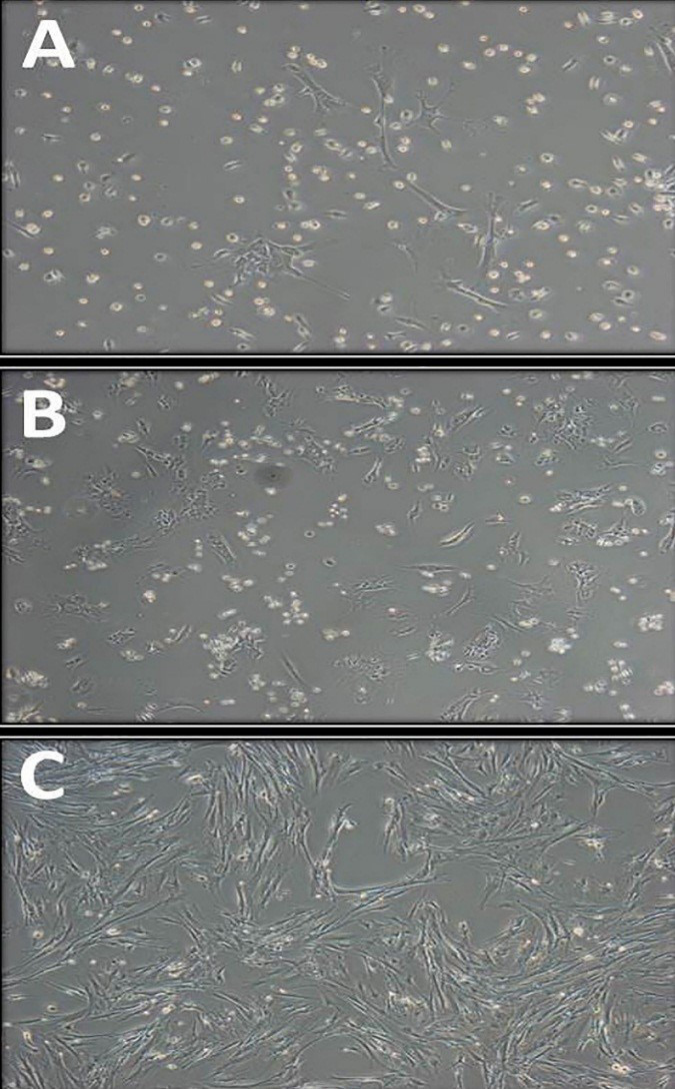
**Mesenchymal stem cells in different passages.** In the first passage after 48 hours, MSCs can be observed, but of little purity (A). In the subsequent passages, the purity of the MSCs gradually increases, and in some cases the cells in the third or fourth passage enter the late phase, in which case the cells lose their duct shape and stop duplication (B). After late period, MSCs replicate rapidly and their purity is also very high (C). (Photos taken with a magnification of 100x)

Purification and proliferation of MSCs is one of the most important steps in working on these cells. If the purified cells do not have sufficient purity or have lost their reproduction and differentiation properties during the purification steps, the results obtained from them will not be very reliable. Figure 1 shows mouse MSCs in different passages. In the first passage, cell clones were formed, but cellular impurities were high. By performing subsequent passages, other cells began apoptosis, were removed from the plate, but the MSCs remained attached to the plate. As a result, the amount of impurities in the cells was reduced and the MSCs became purified after approximately 5 times passaging. In the third or fourth passage, cells in some flasks entered the period of proliferation, and this period was sometimes continued for up to 3 weeks. After this stage, some of the MSCs that were spread out, were spindly shaped, and were quickly replicated (Figure 1).

### Purity of MSCs, DCs and T cells

**Figure 2:**
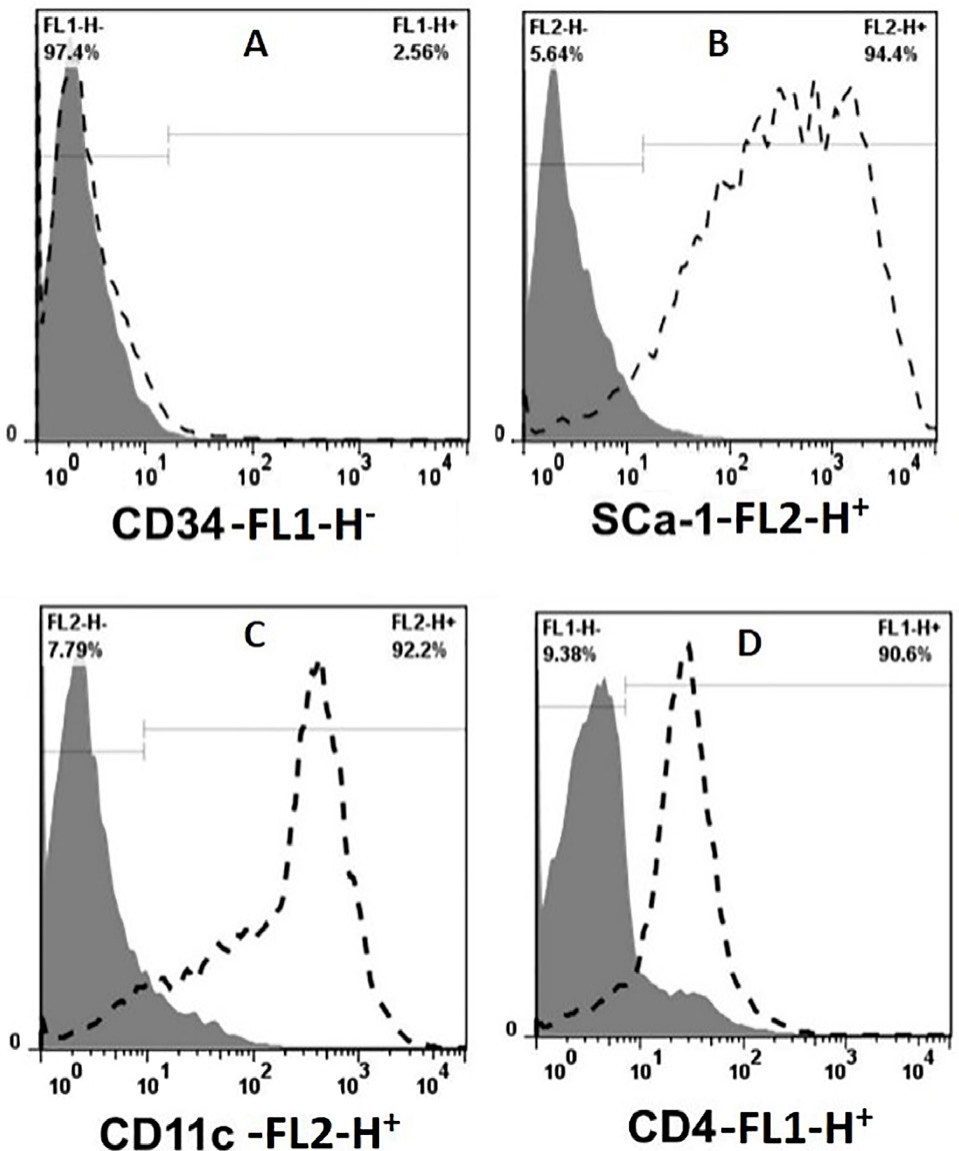
**Evaluation of the purity of MSCs and DCs and T cells by flow cytometry technique.** Using antibodies, purity of MSCs (A and B), DCs (C) and T cells (D) were examined by flow cytometry. The results showed that all 3 cell types have a purity of more than 90%. (Dot Chart: specific antibodies, Solid Chart: Isotype antibody).

The purity of MSCs was evaluated by anti-SCa-1 and CD34 antibodies. The presence of the marker of MSCs (SCa-1) and the absence of specific markers of hematopoietic cells (CD34) in approximately 95% of MSCs showed a high purity of these cells in the fifth passage (Figure 2). Additionally, the purity of DCs and T cells were investigated, respectively, by anti-CD11c and CD4 antibodies. The purity of these cells was also more than 90% (Figure 2).

### Differentiation of MSCs into fat and bone cells

**Figure 3:**
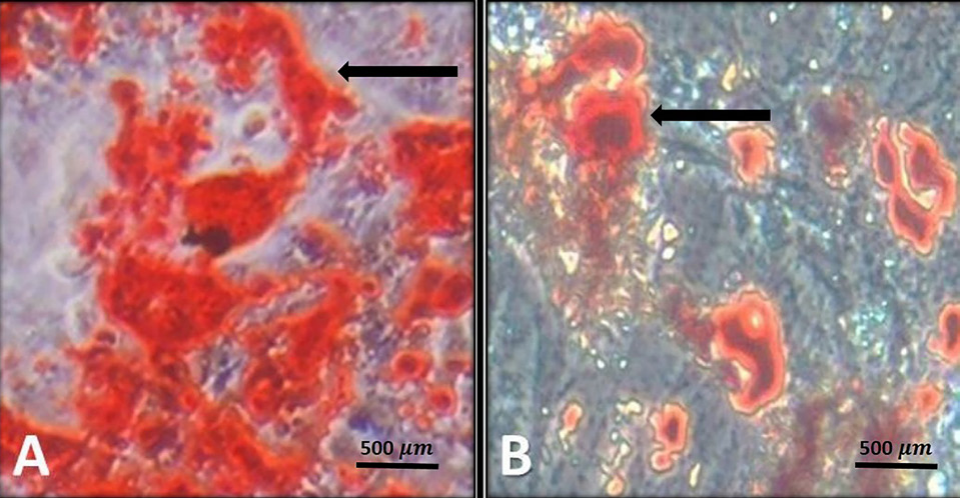
**Differentiation of MSCs into fat and bone cells.** In order to assure the mesenchymal nature of the cell and to evaluate their differentiation power, a differentiation test to bone and fat cells was used. The medium of the cells were then replaced by differentiation into bone and/or fat cells, and cell culture was performed for 14 days. Then, the alizarin red dye was used for bone staining and the oil red dye was used for oil staining. As seen in the figure, MSCs colonies have become red. (A: Mesenchymal cell differentiation into bone cells, B: differentiation of mesenchymal cells into fat cells) (The scale bar represents 500 µm).

MSCs were able to transform into fat and bone cells through cultivation in specific differentiation environments. This characteristic shows that MSCs still retain their distinct property in multiple cell passages (Figure 3).

### Effect of MSCs on expression of mRNA and protein of IDO and Qa2 genes in DCs

**Figure 4:**
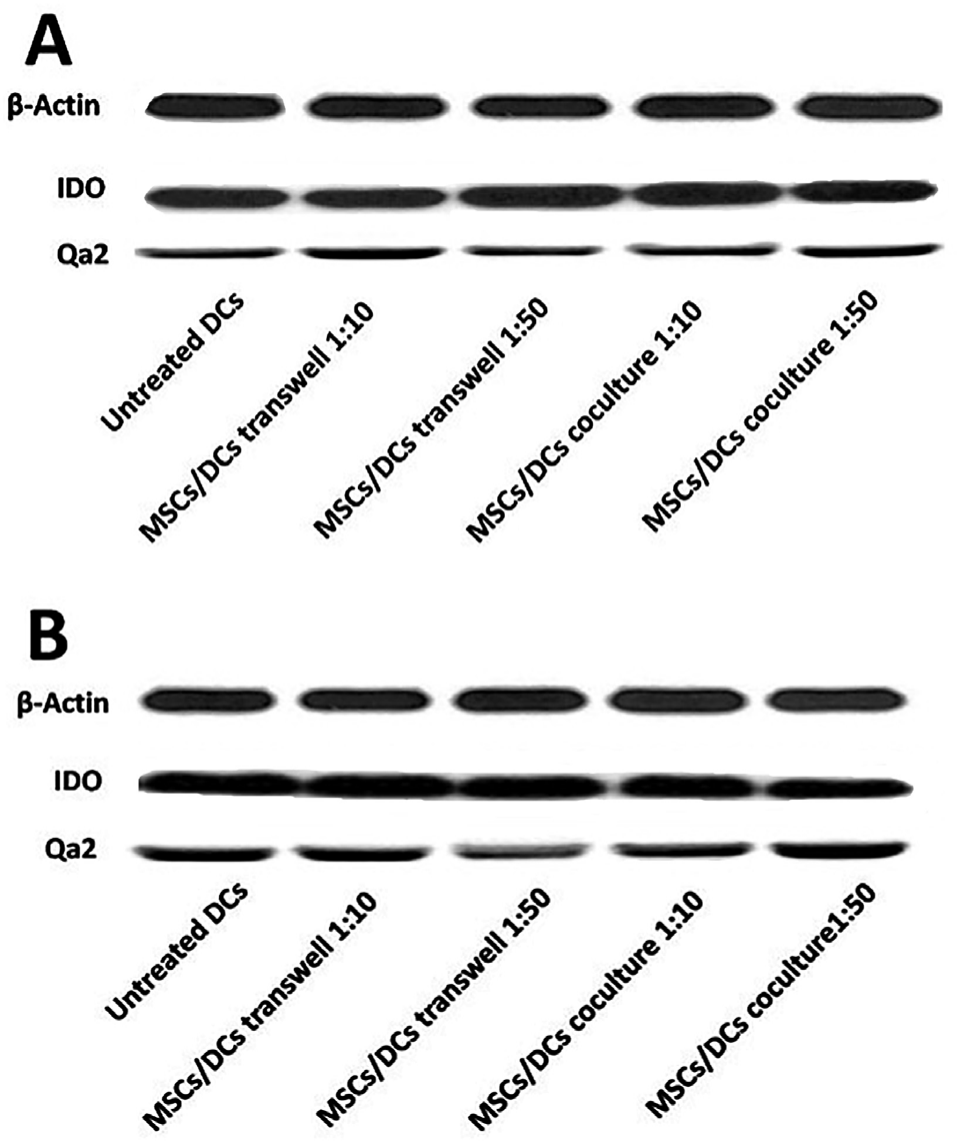
**IDO and Qa2 protein expression in DCs:** IDO and Qa2 protein expression in DCs evaluated by Western Blot. For this purpose, proteins were extracted using RIPPA buffer. Vertical electrophoresis of proteins was performed using SDS-PAGE technique. Subsequently, transfer of proteins from gel to PVDF membrane was carried out using semi-dry western blot set. Then detection of B-actin, IDO and Qa2 proteins were performed by specific antibodies and visualization of the bands was performed by ECL substrate.

**Figure 5:**
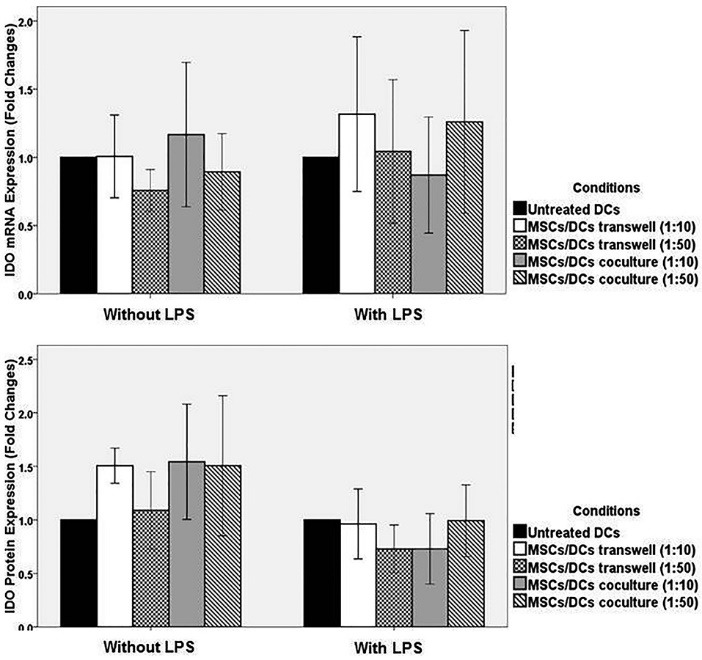
**Effect of MSCs on the expression of IDO gene.** The expression of IDO gene in DCs treated with MSCs were investigated by Real-time PCR and Western Blotting techniques. The expression of IDO protein in DCs that were co-cultured with MSCs (in 1:10 and 1:50 ratios) in absence of LPS was increased, although they were not statistically significant (*P* values: 0.24 and 0.18, respectively). There was no significant difference in expression of IDO mRNA between the studied groups.

**Figure 6:**
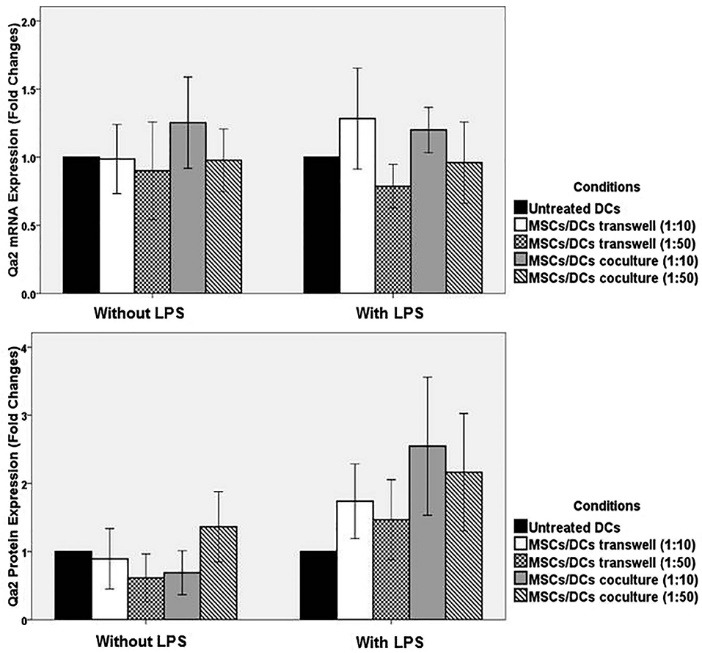
**Effect of MSCs on the expression of Qa2 gene.** The expression of Qa2 gene in DCs treated with MSCs were investigated by Real-time PCR and Western Blotting techniques. The expression of Qa2 protein in DCs that were co-cultured with MSCs (in 1:10 and 1:50 ratios) in presence of LPS was increased, although they were not statistically significant (P-values: 0.09 and 0.33, respectively). There was no significant difference in expression of Qa2 mRNA between the studied groups.

IDO and Qa2 expression were investigated in DCs treated with MSCs by real-time PCR and western blot. In the absence of LPS, a very low level of expression of IDO and Qa2 mRNA were observed in the studied groups. The expression of mRNA of IDO and Qa2 molecules in DCs directly cultured with MSCs in 1: 50 and 1:10 ratios, and DCs of the control group did not significantly differ (*P* > 0.05). In addition, the expression of mRNA of IDO and Qa2 in DCs treated with MSCs in Transwell and control group did not have a significant difference (*P* > 0.05) (Figures 4-6).

The expression of IDO protein in DCs that were co-cultured with MSCs (in 1:10 and 1:50 ratios) in absence of LPS was increased, although they were not statistically significant (*P* values: 0.24 and 0.18, respectively). Furthermore, the expression of Qa2 protein in DCs that were co-cultured with MSCs (in 1:10 and 1:50 ratios) in presence of LPS was increased, although they were not statistically significant (*P* values: 0.09 and 0.33, respectively) (Figures 4-6).

Previous studies have shown that MSCs can inhibit immune cells.^20,33-35^ But, the underlying immunomodulatory mechanisms of MSCs on the cells of immune system is not completely understood. In the present study, we decided to further clarify the mechanisms involved in inducing tolerogenic potency in DCs by MSCs. In addition, it was examined whether the effect of MSC suppression on DCs is directly related to cell-to-cell contact, or only by mediating soluble factors secreted from mesenchymal cells. To achieve this, DCs were directly cultured on MSCs and cultured separately in a Transwell system. In this study, we also examined whether DC maturation factors such as LPS are required to alter DCs to tolerogenic DCs (TolDCs) under the influence of MSCs. In order to achieve this, we have made conditions with LPS or without LPS. The results of this study showed that a low level of mRNA and protein of IDO and Qa2 were expressed in DCs cultured with MSCs as well as DCs of the control group, but no significant difference was observed in the study groups.

Generally, the results of previous studies show that the expression of the Qa2 molecule at the surface of DCs leads to tolerance of immunity. According to our knowledge, there is no published article regarding the expression of IDO and Qa2 in DCs treated with MSCs to compare them to our results. However, there are some articles which studied the effect of MSCs on the expression of other tolerogenic and/or immunogenic molecules in DCs. In this regard, in our previous study, we treated DCs with MSCs and expression of ILT3 on DCs was evaluated. We did not find any differences in ILT3 expression between MSCs treated DCs and untreated ones.^36^ In another study, we showed that PD-L1 expression was higher in DCs treated with MSCs in comparison to the untreated DCs in the presence of LPS.^7^ In another study, the immunomodulatory function of mice MSCs culture supernatant on DCs was studied, and the maturation of DCs was evaluated. Their data reviled that the MSCs culture supernatant down regulated the expression of CD86 co-stimulatory molecule as well as MHC-II on DCs, while the expression of CD40 molecule was not affected by the MSCs culture supernatant. Furthermore they found that, proliferation of T lymphocytes was suppressed by MSCs treated DCs. Additionally, in an MLR, they revealed that secretion of IL-4 cytokine was increased by T cells co-cultured with MSCs treated DCs.^34^ In another study carried out by Hancharou et al, it was shown that human olfactory mucosa-derived MSCs (hOM-MSCs) can significantly increase the expression of both immunogenic (CD86) and tolerogenic (CD85k) markers of DCs.^37^ In another study, it was shown that MSCs supernatant can down-regulate the expression of MHC-II and CD86 in DCs. Besides, the capability of treated DCs to inhibit T cell proliferation, generate Treg cells and cytokines expression in MLR were studied. The results showed that DCs treated with MSCs were able to up-regulate the expression of FOXP3 which is the main marker of Treg cells. Furthermore, T cell proliferation was inhibited in the presence of MSCs treated DCs in MLR assay.^34^ in another study carried out by Krampera et al, it was revealed that BM-derived MSCs have a suppressive effect on T lymphocytes by their cognate peptides in vitro. This suppression affects memory T cells as well as naïve T cells by inhibition of cell proliferation, IFN-γ secretion, and cytolytic function. Furthermore, they found that physiologic tolerance of T lymphocytes is occurred by both deletion and anergy.^38^ In another study, Wang et al demonstrated that co-culture of mature DCs with MSCs in a Transwell system decreased the expression of the CD83, CD80 and CD86 co-stimulatory molecules on DCs, while up-regulated their endocytosis function. They showed that, MSCs treated DCs have functionally impaired T lymphocytes stimulatory function in MLR and shift them from Th1 to Th2 lymphocytes. While the expression of MHC II, CD80 and CD86 molecules were up-regulated on MSCs treated DCs. They suggested that the interaction between MSCs and DCs modulates the function of immune cells.^39^

One of the strength point of this study is this that, there is no published article about the effects of MSCs on the level of expression of the IDO and Qa-2 tolerogenic molecules in DCs which is. On the other hand, one of the weaknesses of this research was that only 24 hours after co- culturing of the DCs and MSCs, the expression of the genes were examined, while the genes were better to be evaluated at 48 hours and 72 hours later, too. Also, in addition to ratios of 1:10 and 1:50, the ratios of 1: 2 and 1: 1 could also be used for MSCs and DCs co-cultures. Additionally, more researches regarding the effect of MSCs on the expression of other genes that contribute to the tolerance of DCs, such as ICOS, ICOS ligand and etc. in MSCs treated DCs seems to be necessary.

## Conclusion

The results of our study rejected the hypothesis that MSCs induce Tol-DCs cells by increasing the expression of IDO and Qa2 gens.

## Ethical Issues

All experiments on mice were performed according to the protocol of the Medical Ethics Committee of the Shiraz University of Medical Sciences and approved by the committee

## Conflict of Interest

The authors report no conflicts of interest.

## Acknowledgments

Our thanks are due to Dr. Maryam Khosravi and Dr. Mehdi Kalani because of their contribution in the process of this research project. It should be noted that the funding of this research project has been provided by Fasa University of Medical Sciences and Transplant Research Center of Shiraz University of Medical Sciences.
